# Low-Intensity Online Intervention for Mental Distress Among Help-Seeking Young People in Hong Kong: A Randomized Clinical Trial

**DOI:** 10.1001/jamanetworkopen.2024.54675

**Published:** 2025-01-15

**Authors:** Yi Nam Suen, Christy Lai Ming Hui, Lauren Ka Shun Lei, Chung Ming Leung, Stephanie Ming Yin Wong, Gabriel Chun Hei Lai, Esther Hang Sze Chau, Michael Tak Hing Wong, Sherry Kit Wa Chan, Gloria Hoi Yan Wong, Eric Yu Hai Chen

**Affiliations:** 1 School of Nursing, Li Ka Shing Faculty of Medicine, The University of Hong Kong, Pokfulam, Hong Kong; 2 Department of Psychiatry, School of Clinical Medicine, Li Ka Shing Faculty of Medicine, The University of Hong Kong, Pokfulam, Hong Kong; 3 Department of Social Work and Social Administration, Faculty of Social Sciences, The University of Hong Kong, Pokfulam, Hong Kong; 4 State Key Laboratory of Brain and Cognitive Sciences, The University of Hong Kong, Pokfulam, Hong Kong; 5 School of Psychology and Clinical Language Science, University of Reading, Reading, UK; 6 Orygen, The National Centre of Excellence in Youth Mental Health, University of Melbourne, Parkville, Australia

## Abstract

**Importance:**

Mental health issues among young people are increasingly concerning. Conventional psychological interventions face challenges, including limited staffing, time commitment, and low completion rates.

**Objective:**

To evaluate the effect of a low-intensity online intervention on young people in Hong Kong experiencing moderate or greater mental distress.

**Design, Setting, and Participants:**

This randomized clinical trial was conducted from May 12, 2022, to September 22, 2023, in Hong Kong. Participants (aged 12-30 years) were recruited from a community project, self-reported moderate to severe distress (Kessler Psychological Distress Scale score ≥5), and were randomized 1:1 to receive the low-intensity online intervention or self-help tips (waitlist group).

**Intervention:**

The low-intensity online intervention group received 4 weekly 1-on-1 online sessions on stress management, sleep, or problem-solving delivered by trained psychological well-being practitioners. The waitlist group received weekly mental health tips via text messaging.

**Main Outcomes and Measures:**

Primary outcomes were changes in Kessler Psychological Distress Scale scores and depression and anxiety subscale scores of the Depression, Anxiety, and Stress Scale. Secondary outcomes included general stress, overall negative emotions, quality of life, sleep quality, resilience, and self-efficacy. The primary outcome analysis was based on intention to treat with the last observation carried forward approach. Sensitivity analyses were conducted using per-protocol and multiple imputation methods.

**Results:**

Of 332 screened participants, 120 (mean [SD] age, 22.4 [3.4] years; 87 [72.5%] female) were randomized. From baseline to 4 weeks, the low-intensity online intervention group and the control group both saw reductions in scores for depression (mean [SD] difference, 6.0 [7.7] and 4.8 [7.9]; *P* = .17; η_p_^2^ = 0.02), anxiety (mean [SD] difference, 6.0 [7.7] and 3.5 [7.7]; *P* = .07; η_p_^2^ = 0.03), and psychological distress (mean [SD] difference, 3.8 [3.8] and 2.9 [3.8]; *P* = .24; η_p_^2^ = 0.01), but none of these differences were statistically significant. However, the intervention group showed greater reductions in general stress (mean [SD] difference, 7.5 [7.2] vs 4.4 [7.2]; *P* = .02; η_p_^2^ = 0.05), negative emotion (mean [SD] difference, 20.3 [19.2] vs 12.7 [19.2]; *P* = .03; η_p_^2^ = 0.04), and increased resilience (mean [SD] difference, 0.5 [0.6] vs 0.2 [0.6]; *P* = .01; η_p_^2^ = 0.05) compared with the waitlist group. Younger participants and those with lower initial distress experienced greater improvements. The findings were supported by the per-protocol analysis but not by multiple imputation analysis.

**Conclusions and Relevance:**

A low-intensity online intervention did not significantly improve distress, depressive, or anxiety symptoms but showed some potential in reducing general stress and negative emotions and improving resilience. These findings are encouraging but not definitive, and caution is needed due to missing data. These data suggest that a low-intensity online intervention may offer a scalable option for youth mental health.

**Trial Registration:**

ClinicalTrials.gov Identifier: NCT05510453

## Key Points

**Question:**

What are the effects of a low-intensity online intervention in community young people with at least moderate mental distress?

**Findings:**

In this randomized clinical trial of 120 young people in Hong Kong, a low-intensity online intervention did not significantly improve overall distress, depression, or anxiety symptoms compared with a control group. However, the intervention showed encouraging but not definitive results in reducing general stress and negative emotions and enhancing resilience.

**Meaning:**

These findings suggest that a low-intensity online intervention may be a scalable and effective solution for reducing stress and enhancing resilience in youth, although further research should minimize missing data and further examine the impact of dropout factors.

## Teaser

This randomized clinical trial evaluates a low-intensity online intervention designed for young people in Hong Kong with mental distress.

## Introduction

Mental disorders, particularly mood and psychotic disorders, contribute significantly to global health burdens, with Hong Kong being no exception. These disorders often emerge during adolescence and have lifelong impacts, with 75% manifesting before the age of 25 years.^1^ They disrupt development, impair quality of life, and hinder community participation, contributing to one-third of global productivity loss.^2,3,4,5^ Early intervention is crucial to mitigating these adverse outcomes, including impaired education, health issues, and entrenched symptoms.^6,7^ Studies show that even early symptoms can significantly burden individuals, families, and society.^5,8,9^ Depressive and anxiety symptoms in youth are often precursors to severe psychopathology in adulthood.^10^ However, many young people face barriers to accessing support, such as stigma and insufficient mental health services. Digital mental health interventions have emerged to bridge these gaps, offering promising solutions to reach underserved populations.^11^

Despite strong evidence supporting the need for early intervention, health care systems often prioritize severe cases, leaving early symptoms underaddressed. This service gap exacerbates stigma and delays treatment,^12^ further increasing the societal burden.^13^ The need for accessible, scalable interventions that prioritize convenience and reduce access barriers is critical.^14^ Low-intensity interventions, characterized by minimal specialist involvement and cost-effective implementation, provide a promising solution.^15^ These interventions focus on self-help and self-management, often led by nonspecialists,^16,17^ yet have shown to be effective in alleviating mental distress.^18^ Young people, in particular, have shown a strong preference for online mental health services^19^ due to the accessibility and flexibility of these services.^20^

Low-intensity online interventions, which vary in duration from a few minutes^21,22^ to more than 10 hours,^23^ use diverse psychological techniques, such as cognitive behavioral therapy,^24,25,26^ acceptance and commitment therapy,^27^ and motivational interviewing.^28,29^ Previous work has demonstrated their efficacy in both in-person and online formats, although fewer studies have focused on the latter.^27^ A challenge for these interventions is the need for therapists with significant training. Implementing complex interventions consistently can be difficult, especially with less experienced staff, which can lead to variability in outcomes. Simplifying intervention language and reducing jargon can help ensure accessibility for all levels of personnel, thereby maintaining treatment efficacy.^30^

This study evaluates a low-intensity online intervention designed for young people in Hong Kong. The intervention targets young adults (aged 12-30 years), a critical developmental period marked by identity formation and role transitions.^31,32^ Navigating challenges related to education, relationships, and living situations often leads to mental health difficulties, particularly mood and anxiety disorders. Despite their unique needs, this demographic is frequently overlooked in mental health services. Accessible interventions, such as a low-intensity online intervention, are essential for addressing mental health disruptions and preventing future disability in this group.

Developed based on a general stress management approach during the COVID-19 pandemic, this low-intensity online intervention was delivered by trained psychology graduates, known as psychological well-being practitioners (PWPs). These practitioners received relatively short training but were supervised by clinical psychologists, ensuring both accessibility and professional oversight. Preliminary data suggest that this low-intensity online intervention could improve young people’s overall psychological well-being.^33^ We hypothesized that participants in the low-intensity online intervention group would experience greater reductions in depressive, anxiety, and distress symptoms, as well as improvements in quality of life, sleep, resilience, and self-efficacy, compared with a waitlist control group. Additionally, we hypothesize that specific subgroups, based on demographics or distress levels, may benefit differently from the intervention, warranting a more nuanced understanding of its effectiveness.

## Methods

This randomized clinical trial (RCT) protocol was approved by The University of Hong Kong/Hospital Authority Hong Kong West Cluster Institutional Review Board. All participants provided written informed consent online, with ample time to ask questions before enrollment. For individuals younger than 18 years, parental consent was obtained. The study adhered to the Consolidated Standards of Reporting Trials (CONSORT)–EHEALTH guidelines for reporting electronic and mobile health interventions.^34^ The trial protocol is provided in Supplement 1.

### Study Design

**Figure 1.**
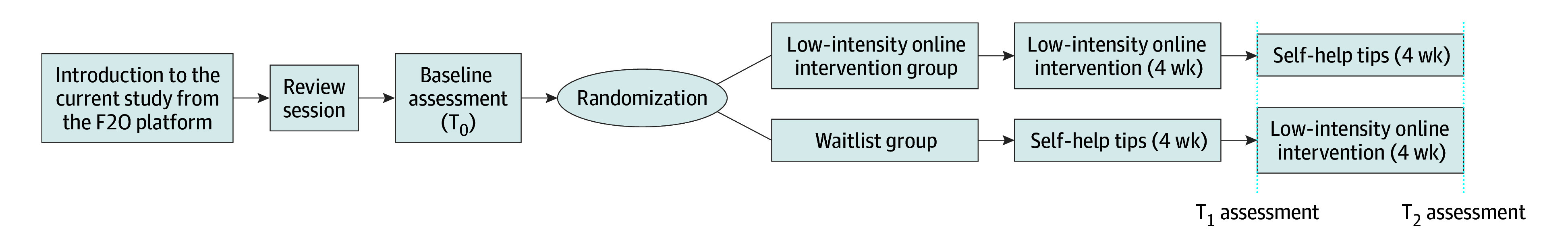
Study Design F2O indicates face-to-face, 1-on-1; T_1_, week 4; T_4_, week 8.

This RCT compared the effects of the low-intensity online intervention with a waitlist control group, in which participants received self-help tips via text messaging (WhatsApp; Meta). Participants were randomized in a 1:1 ratio using a randomizer function (Qualtrics), with block sizes of 4, 6, and 8 to ensure allocation concealment. Participants in the intervention group received 4 weekly online sessions, whereas control participants received weekly self-help materials but no low-intensity online intervention during the waiting period (Figure 1). Given the nature of psychological interventions, participant blinding was not feasible.

### Participants

Between May 12, 2022, and September 22, 2023, participants were recruited via the face-to-face, 1-on-1 (F2O) platform of a territory-wide community youth mental health project named LevelMind@JC.^35^ The F2O platform provides young people with free informal mental health advice from psychiatrists or clinical psychologists.^36^ Eligible participants were aged 12 to 30 years, reported experiencing moderate to severe mental distress (as indicated by a score of ≥5 on the Kessler Psychological Distress Scale),^37^ and had sufficient Chinese proficiency to follow instructions. They also needed reliable internet access for video-based sessions. Exclusion criteria included current psychological treatment, recent suicidal ideation or attempts, inability to provide emergency contact, residence outside Hong Kong, and history of organic brain disorders (eg, epilepsy and brain injury), psychosis, or learning disabilities (eg, special school attendance).

### Interventions

At the time of the clinician’s assessment, eligible participants underwent an online review session via a video chat platform (Zoom; Zoom Video Communications Inc) conducted by trained PWPs to confirm their readiness and eligibility. The PWPs had psychology or mental health backgrounds and received supervision from clinical psychologists. In total, 18 PWPs were involved in the study, serving as research assistants on the team. The group consisted of 11 women and 7 men aged 23 to 31 years; 12 held bachelor’s degrees, and 6 held master’s degrees. The intervention consisted of 3 modules: (1) stress management, (2) sleep and relaxation, and (3) problem-solving skills. Each module involved 4 weekly 1-on-1 sessions (45-60 minutes each) delivered via video meeting. Although participants could complete multiple modules, this study analyzed only data from their first selected module. The low-intensity online intervention and the PWP training were detailed in a previous study.^33^

The modules targeted various psychological techniques. The stress management module focused on identifying stressors, understanding stress mechanisms, and exploring coping strategies. The sleep and relaxation module emphasized sleep hygiene, relaxation techniques, and guided imagery. The problem-solving module used goal-setting models, such as SMART (specific, measurable, achievable, relevant, and time-bound) and SWOT (strengths, weaknesses, opportunities, and threats), to enhance problem-solving abilities. The PWPs followed structured presentations in PowerPoint (Microsoft Corporation) during sessions and provided participants with cognitive behavioral therapy–informed worksheets for practice. Control group participants received weekly self-help tips via text messaging, covering strategies to improve mental health, such as breathing exercises, mindfulness techniques, sleep hygiene tips, and reducing screen time. These tips were general and did not involve direct interaction or personalized feedback (eTable 1 in Supplement 2). The quality of the intervention was assured through regular fidelity checks conducted by the clinical psychologists in the research team. Details can be found in eTable 2 in Supplement 2 and the eMethods in Supplement 2.

### Assessments

**Figure 2.**
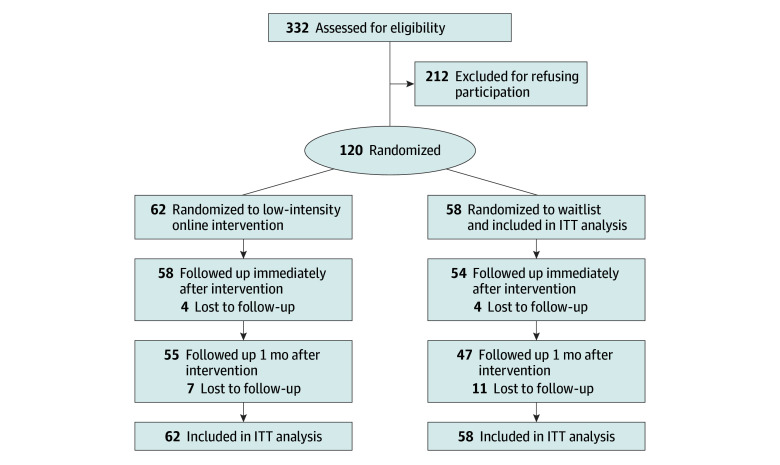
Study Flow CONSORT-EHEALTH Diagram CONSORT indicates Consolidated Standards of Reporting Trials; ITT, intention to treat.

Participants were assessed at (1) baseline, ie, after randomization and prior to the intervention for the low-intensity online intervention group or waiting for the waitlist group (T_0_); (2) after low-intensity online intervention for the intervention group or waiting for the waitlist group (at week 4 [T_1_]); and (3) 1 month after the intervention for the intervention group or waiting for the waitlist group (at week 8 [T_2_]). Participants were tracked at screening, enrollment, allocation, and follow-up in accordance with the CONSORT-EHEALTH reporting guideline (Figure 2).

The prespecified primary outcome measures were the change in scores for the depression and anxiety subscales of the 21-item version of the Depression, Anxiety, and Stress Scale (DASS-D and DASS-A, respectively)^38,39^ and Kessler Psychological Distress Scale scores between baseline and T_1_ assessment in an intention-to-treat (ITT) analysis. Secondary outcomes included changes between baseline and T_1_ assessment in the following self-reported measures: (1) general stress as assessed using DASS-S; (2) overall negative emotion as indicated by the total DASS score; (3) subjective sleep quality assessed as a single item in response to the question, “How was your overall sleep quality in the past week?” from the Pittsburgh Sleep Quality Index^19^; (4) resilience as assessed using the six-item Brief Resilience Scale (BRS)^40^; (5) self-efficacy as assessed using the 2 items “I can remain calm when facing difficulties because I can rely on my coping abilities” and “It is easy for me to stick to my aims and accomplish my goals” from the Generalized Self Efficacy Scale^41^; and (6) health-related quality of life as assessed using the Short Form 6 Dimensions, a 6-dimensional health state classification derived from the 12-item Short Form Health Survey.^42^ To reduce the burden on participants during assessments, clinical psychologists in the research team selected only 1 to 2 items of the Pittsburgh Sleep Quality Index and Generalized Self Efficacy Scale.

### Statistical Analysis

Data were analyzed from October 1, 2023, to June 24, 2024. The sample size calculation was based on the effect size (0.4) of the pilot study.^33^ Allowing for a type I error rate of .05, power of 80%, and allocation ratio of 1:1, using repeated-measures, within-between interaction *F* test, the required sample size for determining a significant group difference between the low-intensity online intervention and waitlist groups was 104 (ie, 52 participants in each group). After accounting for a 15% attrition rate, the target sample size for this trial was 120 participants.

All statistical analyses were conducted using SPSS software, version 25.0 (SPSS Inc), with the significance level set at a 2-sided *P* < .05. Distributions of baseline characteristics were compared between the low-intensity online intervention and waitlist groups with 2-sided *t* tests (continuous measures) and χ^2^ tests (categorical measures). Primary and secondary outcome measures included in the analysis were assessed for normality and outliers. The ITT analysis was performed by carrying the last observation forward approach for the missing data. Repeated-measures analysis of covariance was then used to examine the interaction effect of group (intervention and waitlist groups) × time (from T_0_ to T_1_) with the covariates of age and sex on all the outcome measures. The sensitivity analysis using the per-protocol approach and multiple imputation and the subgroup analysis are detailed in the eMethods in Supplement 2.

## Results

A total of 332 young people who used the F2O service were assessed for eligibility, of whom 120 consented to participate in the RCT (participation rate, 36.1%) (Figure 2). Participants had an mean (SD) age of 22.4 (3.4) years; 87 (72.5%) were female and 33 (27.5%) were male. The demographics and clinical characteristics and retention rate (93% immediately after the intervention and 85% at 1 month after the intervention) of the low-intensity online intervention and waitlist groups were comparable (Table). Participants completed a mean (SD) of 3.3 (1.4) sessions of the intervention, with 94 (78.3%) completing all 4 sessions.

**Table.** Baseline Demographics and Clinical Characteristics of the Study Sample

Characteristic	Mean (SD)^a^		Statistical comparison
	Low-intensity online intervention (n = 62)	Waitlist (n = 58)	
Age, y	22.3 (3.1)	22.5 (3.7)	1768.0^b^
Sex, No. (%)			
Female	45 (72.6)	42 (72.4)	0.000^c^
Male	17 (27.4)	16 (27.6)	
Sleep quality score	2.66 (0.51)	2.57 (0.68)	1701.5^b^
Resilience score	3.70 (0.53)	3.65 (0.56)	1703.0^b^
Self-efficacy score	3.19 (0.50)	3.11 (0.53)	1666.5^b^
DASS-D score	16.45 (8.95)	16.66 (9.57)	1781.5^b^
DASS-A score	15.16 (8.44)	13.72 (7.61)	0.978^d^
DASS-S score	22.45 (7.80)	19.86 (7.42)	1.861^d^
DASS total score	54.06 (21.14)	50.24 (20.57)	1.003^d^
K6 score	10.56 (4.55)	10.34 (4.37)	0.269^d^
HRQOL (SF-6D score)	0.68 (0.11)	0.70 (0.10)	1610.5^b^

Abbreviations: DASS-A, anxiety subscale of the Depression, Anxiety, and Stress Scale; DASS-D, depression subscale of the Depression, Anxiety, and Stress Scale; DASS-S, stress subscale of the Depression, Anxiety, and Stress Scale; HRQOL, health-related quality of life; K6, 6-item Kessler Psychological Distress Scale; SF-6D, Short Form 6 Dimensions.

^a^ Unless otherwise indicated.

^b^ Using the Mann-Whitney *U* test.

^c^ Using the χ^2^ test.

^d^ Using the independent 2-sample *t* test.

### Primary Outcomes

**Figure 3.**
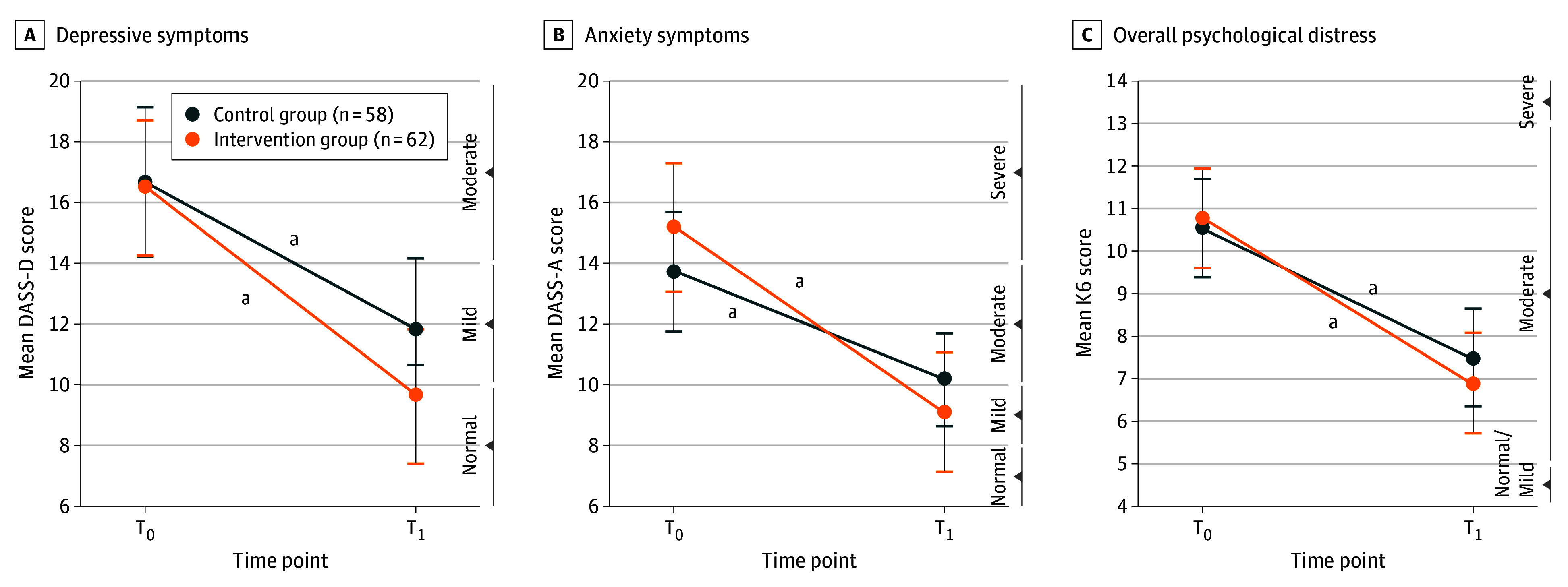
Intention-to-Treat Comparison Between the Low-Intensity Online Intervention and Waitlist Groups on Primary Outcomes All models were adjusted for sex and age. Error bars denote SEs. All interaction effects were not statistically significant: *F*_1,116_ = 1.86, *P* = .17, η_p_^2^ = 0.02 for depressive symptoms, *F*_1,116_ = 3.26, *P* = .07, η_p_^2^ = 0.03 for anxiety symptoms, and *F*_1,116_ = 1.41, *P* = .24, η_p_^2^ = 0.01 for overall psychological distress. DASS-A indicates anxiety subscale of the Depression, Anxiety, and Stress Scale; DASS-D, depression subscale of the Depression, Anxiety, and Stress Scale; K6, Kessler Psychological Distress Scale; T_0_, baseline; T_1_, week 4.

Both the low-intensity online intervention and waitlist groups, respectively, demonstrated significant improvement from T_0_ to T_1_ in depressive symptoms (mean [SD] difference, 6.8 [7.9] and 4.8 [7.9]; *P* = .17; η_p_^2^ = 0.02), anxiety symptoms (mean [SD] difference, 6.0 [7.7] and 3.5 [7.7]; *P* = .07; η_p_^2^ = 0.03), and overall psychological distress (mean [SD] difference, 3.8 [3.8] and 3.8 [2.9]; *P* = .24; η_p_^2^ = 0.01). The improvement in the intervention group was numerically greater than in the waitlist group, but the difference was not statistically significant (Figure 3; eTable 3 in Supplement 2). The nonsignificant findings were also consistent when using both the per-protocol (eTable 4 in Supplement 2) and multiple imputation methods (eTable 5 in Supplement 2).

### Secondary Outcomes

**Figure 4.**
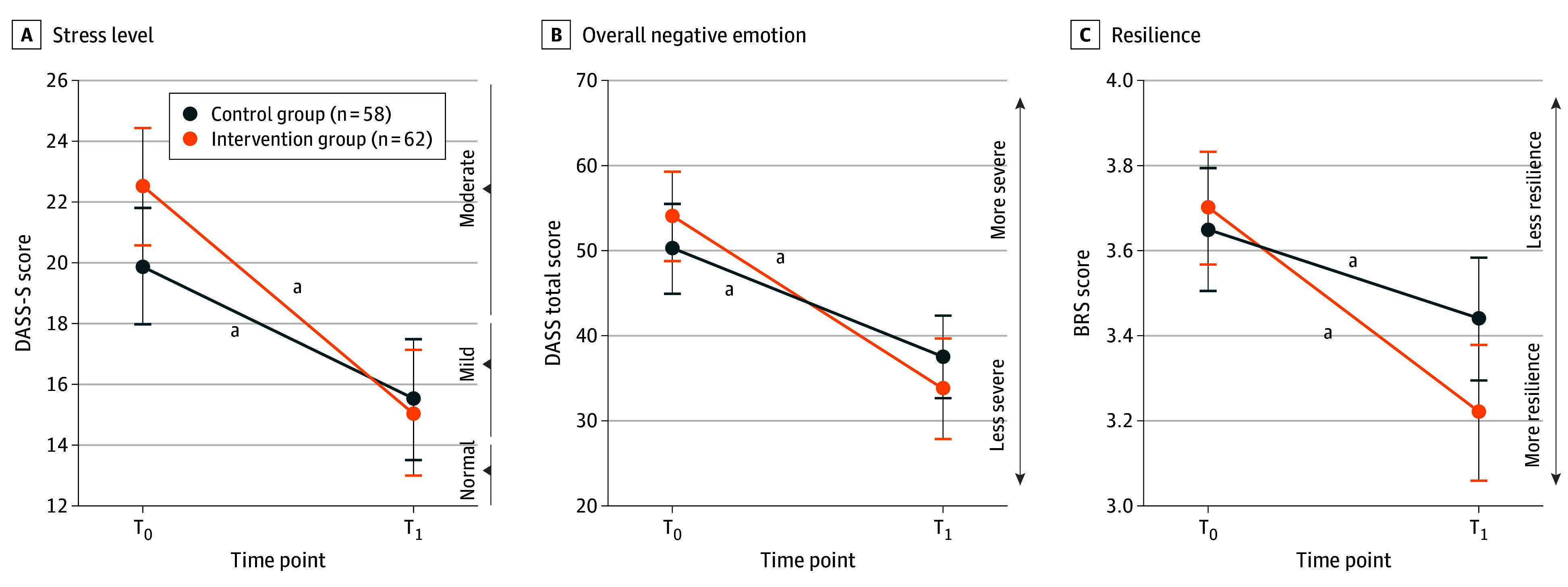
Intention-to-Treat Comparison Between the Low-Intensity Online Intervention and Waitlist Groups on Secondary Outcomes Only models with significant interaction effect are shown. All models were adjusted for sex and age. Error bars denote SEs. All interaction effects were statistically significant: *F*_1,116_ = 5.54, *P* = .02, η_p_^2^ = 0.05 for general stress level, *F*_1,116_ = 4.72, *P* = .03, η_p_^2^ = 0.039 for negative emotion, and *F*_1,116_ = 6.24, *P* = .01, η_p_^2^ = 0.05 for resilience. BRS indicates Brief Resilience Scale; DASS, Depression, Anxiety, and Stress Scale; DASS-S, stress subscale of the Depression, Anxiety, and Stress Scale; T_0_, baseline; T_1_, week 4. ^a^*P* < .001.

The low-intensity online intervention group demonstrated significantly greater improvement than the waitlist group, respectively, in general stress (mean [SD] difference, 7.5 [7.2] vs 4.4 [7.2]; *P* = .02; η_p_^2^ = 0.05), overall negative emotion (mean [SD] difference, 20.3 [19.2] vs 12.7 [19.2]; *P* = .03; η_p_^2^ = 0.04), and resilience (mean [SD] difference, 0.5 [0.6] vs 0.2 [0.6]; *P* = .01; η_p_^2^ = 0.05) (Figure 4; eTable 3 in Supplement 2). Only the significant finding on resilience was also observed in the per-protocol analysis (eTable 4 in Supplement 2), whereas none were found in the multiple imputation analysis (eTable 5 in Supplement 2).

Furthermore, both the intervention and waitlist groups demonstrated significant improvement in self-efficacy and health-related quality of life from T_0_ to T_1_. The improvement in the intervention group was numerically greater than the waitlist group but did not reach statistical significance (eTable 3 in Supplement 2). Only the low-intensity online intervention group demonstrated significant improvement in subjective sleep quality (eTable 3 in Supplement 2), and no interaction effect was observed. These observations were supported by both the per-protocol (eTable 4 in Supplement 2) and multiple imputation analyses (eTable 5 in Supplement 2). The findings of the sensitivity and subgroup analyses are detailed in the eResults and eTables 6 to 9 in Supplement 2. There were no important harms attributed to study participation. No unintended effects were reported by any of the participants.

## Discussion

This RCT assessed the effects of a low-intensity online intervention for young people experiencing moderate to severe mental distress in Hong Kong. The results showed that a low-intensity online intervention, delivered by trained PWPs, did not significantly improve distress, depressive, or anxiety symptoms. However, the intervention demonstrated some potential benefits in secondary outcomes, including the reduction in general stress and negative emotions and enhancement in resilience. Such findings should be interpreted cautiously because they became statistically nonsignificant after multiple testing corrections.

Although the intervention and control groups both exhibited numerical improvements in distress, depressive, and anxiety symptoms, the differences failed to reach statistical significance, indicating that the intervention did not significantly outperform the control group in these areas. The absence of difference may be due to several factors, including the relatively short intervention period (4 weeks) and the moderate level of baseline distress among participants, which made improvements more difficult to detect. Previous research suggests that low-intensity interventions may require longer durations to produce significant effects on more entrenched symptoms, such as depression and anxiety.^43^ Furthermore, the paraprofessional delivery model may have limited the depth of therapeutic engagement, as more complex cases might require specialized care, even within low-intensity frameworks.^44^

Our findings are consistent with broader literature on the effectiveness of low-intensity psychological interventions in reducing general distress and improving well-being.^45^ Although prior work has reported significant reductions in depressive and anxiety symptoms with low-intensity interventions,^46^ the lack of significant findings for these outcomes suggests that further investigation is needed to determine the specific conditions under which a low-intensity online intervention may be most effective for alleviating severe symptoms. This could involve examining the intervention’s duration, intensity, or content modifications.

Another possible reason for the nonsignificant finding may be the notable improvement observed in the control group, potentially attributable to various factors, which resulted from the self-help tips delivered. Self-help interventions can enhance self-efficacy by giving individuals a sense of control over their well-being. Their accessibility and flexibility allow for consistent engagement, and many incorporate cognitive and behavioral strategies. Additionally, participants may have experienced expectancy effects, anticipating positive outcomes simply by engaging with the material.

Although primary outcomes yielded nonsignificant results, the low-intensity online intervention showed some potential benefits in secondary outcomes, such as general stress, negative emotion, and resilience. These benefits could be attributed to its structured online format and the emphasis on stress management, sleep, and problem-solving, contrasted with the generic self-help advice provided to the control group. These effects were particularly pronounced in younger participants and those with lower baseline distress, highlighting the potential benefit of early mental health interventions for these groups. Future larger studies should provide a more detailed explanation. The use of young PWPs, who were close in age to the participants, may have fostered a sense of trust and empathy, making it easier for participants to engage with the intervention and open up about their mental health challenges.

The significant improvement in resilience aligns with research showing that online interventions enhance coping skills in vulnerable youth.^47^ The low-intensity online intervention equips participants with practical tools for managing stressors, likely contributing to these outcomes. This finding is especially relevant given the increasing demand for scalable, low-cost interventions that can be delivered with minimal resources.

Overall, caution is warranted in interpreting these secondary findings due to their nonsignificance after multiple testing corrections and observed inconsistencies in the multiple imputation analysis. Although the primary ITT and per-protocol analyses supported the main findings, the multiple imputation analysis did not corroborate these results for some outcomes. This raises concerns about potential biases related to missing data and the limitations of different analytical methods. However, by providing a range of estimates—from the most conservative (last observation carried forward) to the most moderate (multiple imputation) and the maximum possible effects (per protocol)—we aimed to offer a balanced view of the data, allowing readers to critically assess the findings from multiple perspectives.

The low-intensity online intervention, developed for Hong Kong’s youth during the COVID-19 pandemic, highlighted the need for low-barrier, transdiagnostic online mental health services. It addressed the increased mental health demand among young people facing unique pandemic challenges, offering continuous psychological support despite mobility restrictions.^48^ Future evaluations of the cost-effectiveness of low-intensity online interventions could inform the development of similar interventions globally, reflecting their broader societal impact.

### Limitations

Several limitations should be considered when interpreting these findings. First, the study’s low participation rate may limit the generalizability of the results and introduce potential selection bias. Participants who completed the study may differ from those who did not, potentially skewing the findings toward individuals who were more motivated or engaged with the intervention. Second, most participants in this study were female (72.5%), which aligns with research showing that women are more likely than men to seek help for mental health issues; however, the gender imbalance limits the generalizability of our findings, particularly to males. Third, the relatively short follow-up period (1 month) may not have been sufficient to capture the long-term effects of the low-intensity online intervention, particularly for outcomes such as depression and anxiety. Future research should include longer follow-up periods to assess the sustained impact of the intervention. Fourth, although the paraprofessional delivery model enhances scalability and accessibility, it may have limited the intervention’s therapeutic depth for participants with more complex mental health needs. Additionally, the lack of correction for multiple testing raises the risk of type I errors, meaning that some significant findings (eg, for stress, negative emotion, and resilience) could have occurred by chance. However, the appropriateness of multiple testing correction in analyses of discrete outcomes remains debated.^49,50^ We reported exact *P* values to allow readers to interpret the significance within the study’s specific context. Fifth, the inability to mask participants and the reliance on self-reported outcomes may have introduced bias, although validated scales mitigated this concern to some extent.

## Conclusions

The low-intensity online intervention did not significantly improve distress, depressive, or anxiety symptoms, but it did show potential—although not definitive—benefits in reducing general stress and negative emotions and enhancing resilience. These results should be interpreted with caution due to their sensitivity to missing data, reflected in inconsistencies across different analytical approaches. Future research should prioritize better handling of missing data, enhancing participant retention, and tailoring the intervention to specific subgroups, while also exploring its long-term efficacy. In conclusion, our findings indicate that a low-intensity online intervention could provide a scalable solution to mental health challenges in underserved regions.

## Notes

Supplement 1.
Trial Protocol

Supplement 2. **eTable 1.** Details of the Self-Help Tips Delivered via WhatsApp **eTable 2.** Fidelity Assessment and Its Outcomes **eMethods.** Supplementary Methods **eResults.** Supplementary Results **eTable 3.** Statistics for the Intention-to-Treat Comparison Between the LiON and WL Groups on All Outcomes **eTable 4.** Statistics for the Per-Protocol Comparison Between the LiON and WL Groups on All Outcomes **eTable 5.** Statistics for the Multiple Imputation Comparison Between the LiON and WL Groups on All Outcomes **eTable 6.** Subgroup Analysis by Distress Levels on the Intention-to-Treat Comparison Between the LiON and WL Groups on All Outcomes **eTable 7.** Subgroup Analysis by Sex on the Intention-to-Treat Comparison Between the LiON and WL Groups on All Outcomes **eTable 8.** Subgroup Analysis by Age on the Intention-to-Treat Comparison Between the LiON and WL Groups on All Outcomes **eTable 9.** Subgroup Analysis by Modules on the Intention-to-Treat Comparison Between the LiON and WL Groups on All Outcomes

Supplement 3.
Data Sharing Statement
