# Supplementary material for: Low-Intensity Online Intervention for Mental Distress Among Help-Seeking Young People in Hong Kong: A Randomized Clinical Trial
Source: JAMA Netw Open. 2025 Jan 15;8(1):e2454675. doi: 10.1001/jamanetworkopen.2024.54675 (PMC11736507; doi:10.1001/jamanetworkopen.2024.54675)
Supplement: Supplement 1. — Trial Protocol [file jamanetwopen-e2454675-s001.pdf]

Supplement 1. Study protocol.

IRB Application - Study Protocol

1. General Information

1.1 Title

A Randomised Controlled Trial of Low-Intensity Online Intervention (LiON) for Distressed Youths in Hong Kong

2. Background information

Youth marks a period of significant life transitions. Despite being a phase of optimal physical health, young people are often most vulnerable to the development of psychiatric symptoms and disorders. Studies have found up to 75% of mental disorders in adulthood have their onset before the age of 25 (Lin et al., 2008; Kessler et al., 2005), suggesting youth marks a critical period of intervention. Across the various psychiatric conditions, depressive and anxiety disorders have consistently been reported to be two of the most common conditions in young people globally (Merikangas et al., 2010; Racine et al., 2021).

Both depressive and anxiety disorders are related to various negative impacts on youths' lives, such as undermining academic achievements, with other negative influences found in past studies (Birmaher et al., 1996; Fletcher, 2008, Woodward and Fergusson, 2001; Swan and Kendall, 2016). Notably, an increasing number of studies have found not only can clinical disorders, but also early symptoms of such conditions, can cause significant burden on the individual, their family and peers, as well as the larger society (Spencer et al., 2018; Lynch and Clarke, 2006; Wingrove and Rickwood, 2020).

Existing healthcare services, however, tend to prioritise service targeting those with severe mental health needs. Knowledge about subtle mental health symptoms is lacking among the general public, which can contribute to delayed care and treatment (Fung et al., 2021). In addition, mental health services in Hong Kong are not only characterised by long waiting times but are also coupled with significant stigma, which can in turn prevent help-seeking, especially among young people (Sun et al., 2017; Yap, Wright, and Jorm, 2011).

2.1 Mental health needs in Hong Kong

The Hong Kong population has undergone a series of highly stressful events traceable since June 2019, from large-scale social unrest to the local outbreak of Coronavirus disease 2019 (COVID-19) in January 2020, and more recently a fifth wave of COVID-19 cases since February 2022. Local studies have shown significant levels of depressive and anxiety symptoms in the local population resulting from such large-scale population-level stressors and other personal life stressors, particularly in young people (Ni et al., 2020; Wong et al., 2020).

In addition, the city has recently reported daily local positive COVID-19 cases as high as 50,000 during the fifth wave of the epidemic (as of March 2022). Increasingly stricter social distancing policies related to the COVID-19 situation has as a result been put in place, such as compulsory COVID-19 testing, mandatory quarantine at home or specified quarantine centres, temporary closure of certain venues, changes to school schedules, and other changes to work patterns. Health services have either become overwhelmed or suspended under the strain of COVID-19; the waiting time for non-urgent health services has also been further lengthened as a consequence, including mental health services. The majority of in-person interventions and services have also been switched to online modes whenever possible to reduce in-person contacts.

2.2 Significance of low-intensity online-based interventions for young people

In view of the significance of early engagement and intervention for those with mental health needs and the current situation in Hong Kong, the implementation of low-intensity online-based interventions (LiON) for mental health appears to be a largely promising approach.

Low-intensity refers to low usage of "specialist therapist time" (Bower and Gilbody, 2005), or usage in a cost-effective way, such as in a group-based Cognitive Behavioural Therapy (CBT) context). Low-intensity interventions focus on delivering self-help and self-management skills which can be led by non-specialists, which in turn help to reduce the cost required in both training and the delivering of intervention. Recent studies have also increasingly highlighted young people's preference to access mental health services through online platforms (Becker et al., 2016).

Due to limited mental health resources and the high demand for mental health services in Hong Kong, such online-based low-intensity interventions – *when shown to be effective* – can be largely helpful in reducing societal costs, reduce the barriers to help-seeking, and facilitate large-scale implementation of mental health services on a population level.

2.3 The current study

In order to investigate the efficacy of LiON in reducing symptoms of depression, anxiety, and distress in young people, a wait-list control randomised controlled trial (RCT) design will be adopted in this study. The effectiveness of this low cost approach in improving functioning, perceived sleep quality, stress coping abilities, and general self-efficacy will also be examined.

3. Study objectives and purposes

3.1 Study aims

The current study aims to evaluate the effectiveness of LiON, as compared with the control group, in reducing (1) depressive and anxiety symptoms, (2) distress symptoms, (3) perceived stress, as well as in (4) improving functioning, (5) sleep quality, (6) stress coping abilities, and (7) general self-efficacy in young people in Hong Kong.

3.2 Study outcomes

All measures of this study will be conducted at baseline, i.e., pre-intervention (T0), post-intervention (T1), and 1-month follow-up (T2). The time frame will be past month for T0 assessments and past week for both T1 and T2 assessments. Basic demographics will also be collected at baseline.

3.2.1 Primary outcomes

Primary outcomes of this study include improved symptoms of depression, anxiety, and distress at post-intervention (T1) and 1-month follow-up (T2).

Symptoms of depression and anxiety will be assessed using the depression and anxiety subscales (DASS-D and DASS-A, respectively) of the 21-item Depression Anxiety Stress Scale (DASS-21; Lovibond & Lovibond, 1995). The DASS-D and DASS-A each contains 7 items that asks participants about their symptoms of depression and anxiety, respectively, using a 4-point Likert scale (from "did not apply to me at all" [0] to "apply to me very much, or most of the time" [3]). The final score for each subscale was generated by multiplying the sum of the individual items by a value of 2, yielding a score range from 0 to 42, with a higher score indicating higher severity.

Symptoms of distress will be assessed using 6 items from the Kessler Kessler Psychological Distress Scale (Kessler et al., 2002). Items were rated on a 5-point Likert scale (from "none of the time" [0] to "all of the time" [4]) and summed to generate the final distress score. The total score ranges from 0 to 24, with a high score indicating greater distress.

3.2.2 Secondary outcomes

Secondary outcomes of this study include functioning (two items from the Kessler Psychological Distress Scale on days of reduced and lost productivity), perceived sleep quality (1 item from the Pittsburgh Sleep Quality Index [PSQI]), perceived stress level (single item Subjective Level of Stress [SLS-1]), stress coping abilities (Brief Resilience Scale [BRS]) and general self-efficacy (General Self-Efficacy Scale [GSE]). Information about the assessments is summarised in the table provided in **Appendix A**.

3.3 Hypotheses

- Those in the LiON group, as compared to the control group, will show greater reductions in (1) depressive and anxiety symptoms, (2) distress symptoms, and (3) perceived stress.
- Those in the LiON group, as compared to the control group, will also show greater improved (4) functioning, (5) perceived sleep quality, (6) stress coping abilities, and (7) general self-efficacy.

4. Study design, randomisation, and recruitment

4.1 Study design and randomisation

This study will adopt an open labelled wait list RCT design (**Figure 1**). Participants will be randomly assigned using a computer-generated sequence into either the (i) low-intensity online intervention (LiON) group (n = 60) or (ii) wait list control (WLC) group (n = 60) in a 1:1 ratio. This study design was adopted to ensure all participants will be able to receive LiON during the period of this study.

Those randomised to the LiON intervention group will first receive the 4-week LiON, with generic self-help tips provided to each participant before completing the T2 assessment. Meanwhile, those randomised to the WLC group will first receive generic self-help tips for a period of 4 weeks, followed by the 4-week LiON before completing the T2 assessment.

Random blocks will be used for randomisation.

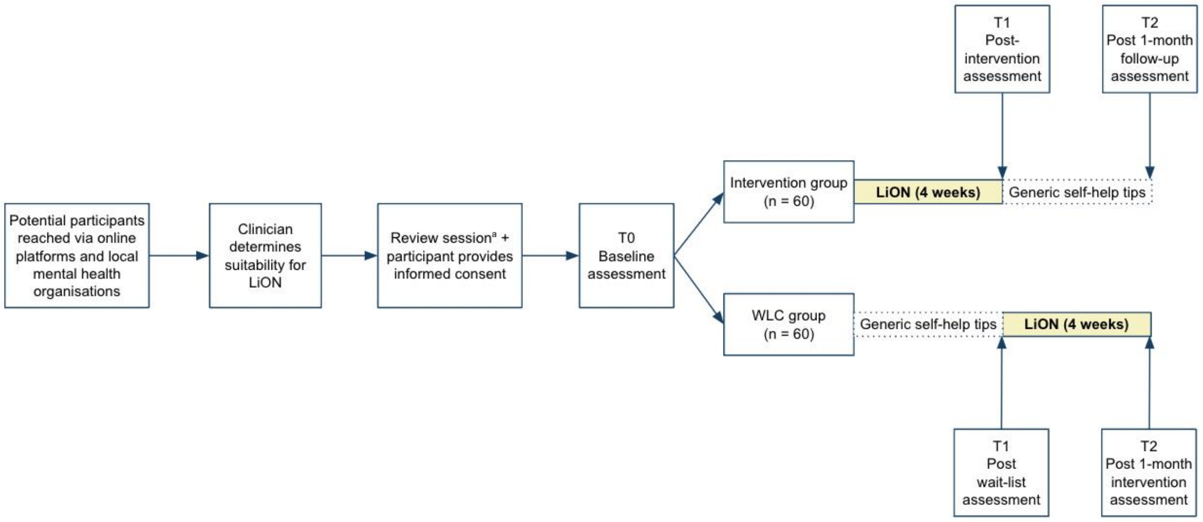

[a]During the review session, the trained interventionist will discuss the concerns and needs of the potential participant to determine the goal of the 4-week LiON sessions, ensure the potential participant meets the inclusion and exclusion criteria of the study, and provide initial briefing about the LiON (including module structure, components of focus).

Figure 1. Flow of the study recruitment and assessment process.

4.2 Details about the process of the RCT and intervention

4.2.1 Before intervention

Prior to the commencement of the LiON, all participants will first be screened for eligibility for participation by a professional clinician of the research team (i.e., psychiatrists or psychologist, senior social worker). An online review session will also be first provided by the trained interventionist (who will be a graduate with background in psychology, social work, or other related mental health disciplines with prior and ongoing training from senior clinicians of the research team) to each participant to determine his or her needs and ensure all inclusion and exclusion criteria are met. Briefing about the LiON, such as module structure and components of focus will also be provided during this session.

4.2.2 The Low-intensity Online Intervention (LiON)

The LiON in the current study is comprised of three core modules identified to be core areas of stress and needs of participants based on clinical experiences of the research team. The three modules include (1) stress management, (2) sleep and relaxation, and (3) problem-solving skills.

Each module consists of four 1-on-1 sessions which will be conducted through online platforms (such as Zoom). Participants will attend these sessions once a week.

All online modules were designed to target different psychological techniques to manage stress, improve sleep quality and relaxation techniques, as well as improve problem-solving skills.

Interventionists will guide participants with the support of powerpoint slides to identify session goals and explain psychological techniques and exercises for practising. Worksheets (likened to "homeworks" in CBT) are also given to participants to perform intervention exercises during the session. A copy of the powerpoint slides and additional worksheets will also be provided to participants.

The content of each module is illustrated as follows:

(1) Stress management:

- This module aims to guide participants in recognising their source of stress, teach the mechanisms underlying stress and importance of identifying different presentations of stress. The main aim of this module is to provide information about the most recommended models of stress management, and exploring healthy coping strategies.

(2) Sleep and relaxation:

- This module focuses on identifying factors impeding participants' sleep and learning relaxation skills. In this module, information on sleep hygiene and relaxation techniques such as guided imagery are provided and practised with participants.

(3) Problem-solving:

- This module is devoted to teaching the importance of dissecting and restructuring problems and doing so by adopting goal-setting models such as SMART model (Strengths, Weaknesses, Opportunities & Threats). Participants will be guided to practice each model with different problems they may face.

4.2.3 Generic self-help tips

All participants will also be provided with generic self-help tips. For those in the LiON intervention group, these self-help tips will be provided after the 4-week LiON. For those in the WLC group, these self-help tips will be provided during the first 4 weeks after randomisation.

These generic self-help tips will include general advice on what one can do to improve their conditions. Content of these tips may include the following:

- Breathing exercises
- Mindfulness-based exercises
- Exercise
- Sleep
- Reduce mobile phone use

4.3 Recruitment and assessment flow

- Subjects will be recruited via invitations from *headwind* F20
- Subjects will first complete an online screening (including the Kessler Psychological Distress Scale to determine distress level). A clinician (e.g., psychiatrist of the research team) will meet the participant through an online platform (such as Zoom) to further conduct a screening of the condition and needs of the participant to determine eligibility and suitability for LiON and the current study.
- Eligible subjects will be given details about the LiON and presented with the informed consent online via Qualtrics.
- For participants below the age of 18, parental or guardian consent would be sought online via Qualtrics before proceeding with the study.
- After consent is sought, participants will be asked to complete the online questionnaire via Qualtrics (at T0), which items are listed on Table 1 , which can be self-administered (a project staff will be available for the process if requested).
- Individual randomisation will be conducted through a computer-generated sequence.
- See **Figure 1** for further details about the flow of recruitment and study design.

4.4 Minimisation and avoidance of bias

Participant blinding is not possible in a psychological intervention study. Randomisation will be carried out via a computer-generated sequence. To minimise differential expectations in the different conditions, descriptions of the different arms of the study will be presented to subjects in a neutral and descriptive language in the online informed consent form.

4.5 Major ethical issues

The study involves participants from a community youth in Hong Kong. The guidelines of handling of personal data such as full name and telephone number will be strictly followed. There is no known hazard or discomfort associated with the semi-structured interviews, online questionnaires via Qualtrics and the intervention, but some participants might not be comfortable with items related to mental illness. Participants are free to withdraw from the study at any tie point without any consequence to their help-seeking psychiatric service. Guardian or parental consent will be required for participants under the age of 18 through online questionnaires via Qualtrics. Information obtained from the consent form will only be accessible by designated research staff. Qualtrics' servers are protected by high-end firewall systems and scans are performed regularly to ensure that any vulnerabilities are quickly found and patched. Application penetration tests are performed annually by an independent third-party. All services have quick failover points and redundant hardware, with backups performed daily.

Referrals to external health services may be made with the consent of the participants or their guardians or parents if participants are screened to have diagnosable mental disorders. The risks and precautions associated will be explained clearly through the disclaimer and consent process and stipulated in the disclaimer and consent form.

5. Study subjects and sample

Eligibility of participants is determined according to the following inclusion and exclusion criteria:

5.1 Inclusion criteria:

Inclusion criteria will be (1) **young people aged between 12–30;** (2) who have sufficient proficiency in Chinese to understand verbal and written instructions and give informed consent; (3) with symptoms of mental distress not specific to depressive or anxiety symptoms.

5.2 Exclusion criteria:

Exclusion criteria will be (1) receiving any forms of cognitive behavioural therapy (CBT), (2) active suicidal ideation/attempts within the past one month, (3) inability to turn on camera for the course of the intervention, (4) inability to provide emergency contact, and (5) located outside of Hong Kong.

6. Sample-size and calculation

The findings of our pilot study (n = 34) suggested that LiON yields an effect size of 0.4 when compared with controls with no intervention at all. To test the main hypothesis that LiON is more effective in reducing participant's distress level than online self-help tips, we estimate the effect size could be halved, with alpha = 0.05 and power = 80%, 52 subjects are needed for each group totalling 104 subjects. After accounting for a 15% attrition, we target 120 subjects for the current study.

7. Statistical Analysis

All statistical analyses will be carried out using IBM® SPSS® with the significance level set at 0.05. Repeated measure ancova model will be used to compare the changes of distress level between two groups with adjustment for the variables that exhibit baseline differences.

A planned analysis will take place after data is available for the first 60 subjects, to provide a more accurate estimate of the power of the study to adjust the final sample size and allocation to the study arms, according to the principles of adaptive trial design.
