# Supplementary material for: Low-Intensity Online Intervention for Mental Distress Among Help-Seeking Young People in Hong Kong: A Randomized Clinical Trial
Source: JAMA Netw Open. 2025 Jan 15;8(1):e2454675. doi: 10.1001/jamanetworkopen.2024.54675 (PMC11736507; doi:10.1001/jamanetworkopen.2024.54675)
Supplement: Supplement 2. — eTable 1. Details of the Self-Help Tips Delivered via WhatsApp eTable 2. Fidelity Assessment and Its Outcomes eMethods. Supplementary Methods eResults. Supplementary Results eTable 3. Statistics for the Intention-to-Treat Comparison Between the LiON and WL Groups on All Outcomes eTable 4. Statistics for the Per-Protocol Comparison Between the LiON and WL Groups on All Outcomes eTable 5. Statistics for the Multiple Imputation Comparison Between the LiON and WL Groups on All Outcomes eTable 6. Subgroup Analysis by Distress Levels on the Intention-to-Treat Comparison Between the LiON and WL Groups on All Outcomes eTable 7. Subgroup Analysis by Sex on the Intention-to-Treat Comparison Between the LiON and WL Groups on All Outcomes eTable 8. Subgroup Analysis by Age on the Intention-to-Treat Comparison Between the LiON and WL Groups on All Outcomes eTable 9. Subgroup Analysis by Modules on the Intention-to-Treat Comparison Between the LiON and WL Groups on All Outcomes [file jamanetwopen-e2454675-s002.pdf]

Supplementary Online Content

Suen YN, Hui CLM, Lei LKS, et al. Low-intensity online intervention for mental distress among help-seeking young people in Hong Kong: a randomized clinical trial. *JAMA Netw Open*. 2025;8(1):e2454675. doi:10.1001/jamanetworkopen.2024.54675

eTable 1. Details of the Self-Help Tips Delivered via WhatsApp

eTable 2. Fidelity Assessment and Its Outcomes

eMethods. Supplementary Methods

eResults. Supplementary Results

eTable 3. Statistics for the Intention-to-Treat Comparison Between the LiON and WL Groups on All Outcomes

eTable 4. Statistics for the Per-Protocol Comparison Between the LiON and WL Groups on All Outcomes

eTable 5. Statistics for the Multiple Imputation Comparison Between the LiON and WL Groups on All Outcomes

eTable 6. Subgroup Analysis by Distress Levels on the Intention-to-Treat Comparison Between the LiON and WL Groups on All Outcomes

eTable 7. Subgroup Analysis by Sex on the Intention-to-Treat Comparison Between the LiON and WL Groups on All Outcomes

eTable 8. Subgroup Analysis by Age on the Intention-to-Treat Comparison Between the LiON and WL Groups on All Outcomes

eTable 9. Subgroup Analysis by Modules on the Intention-to-Treat Comparison Between the LiON and WL Groups on All Outcomes

This supplementary material has been provided by the authors to give readers additional information about their work.

© 2025 Suen YN et al. *JAMA Network Open*.

eTable 1. Details of the Self-Help Tips Delivered via WhatsApp

**28-Day self-help booklet**	
Week1<br><br>**Eye (visual) awareness exercises**	• Exercise<br>1. Blink<br>2. Nature view exposure<br>3. Nature view exposure (advanced)<br>• Summary of skills
Week 2<br><br>**Ear (auditory) awareness exercises**	• Exercise<br>1. Nature sound exposure (forest, stream)<br>2. Surrounding sound exposure<br>3. Healing sound exposure (raindrop, soft music)<br>• Summary of skills
Week 3<br><br>**Taste (gustatory) awareness exercises**	• Exercise<br>1. Various natural colours in eating<br>2. Eating with five senses<br>3. Mindful eating<br>• Summary of skills
Week 4<br><br>**Nose (olfactory) awareness exercises**	• Exercise<br>1. Smell exposure<br>2. Relaxation breathing<br>3. Humming while breathing<br>• Summary of skills

© 2025 Suen YN et al. *JAMA Network Open*.

eTable 2. Fidelity Assessment and Its Outcomes

Module	Full mark	Assessed cases	Achieved marks
Sleep management and relaxation	48	3	47, 47, 48
Stress coping	58	11	50, 53, 54, 54, 55, 55, 55, 56, 56, 56, 58
Problem solving	40	4	32, 35, 37, 38

© 2025 Suen YN et al. *JAMA Network Open*.

eMethods. Supplementary Methods

A sensitivity analysis was conducted using a per-protocol approach, focusing on participants who strictly adhered to the treatment protocol by completing all four sessions of the LiON intervention. Additionally, multiple imputation (MI) was performed to handle missing data, with 50 imputations per missing value. To ensure consistency between the imputation and analysis models, the imputation model included the group assignment, module, sex, age, baseline variables, as well as T_1 and T_2 outcomes (Hayati Rezvan, Lee, & Simpson, 2015).

The subgroup analysis in the study aimed to examine if the observed effects were specific to any particular subgroup of participants. These subgroups were classified based on distress level (low with K6<11 and high distress level with K6≥11), age (younger participants with age less than the median, ranging from 12-21 years, and older participants with age greater than or equal to the median, ranging from 22-30 years), biological sex (female and male), and chosen modules. The classification of distress level was based on the service model of the LevelMind@JC project, where young people with a score of 11 or above were considered as an at-risk group, as mentioned by Hui et al. (2022) This group required more structured psychotherapy, medical treatment, social care, and educational support. On the other hand, those with a K6 score of less than 11 were considered suitable for preventative early intervention.

Fidelity monitoring

The quality of the intervention was assured through regular fidelity checks conducted by the clinical psychologists in the research team. We randomly selected 15% of the cases to assess in which the clinical psychologists observed the PWPs delivering all four intervention sessions of a selected case, and then rate the PWPs' adherence to the intervention protocol using a fidelity assessment checklist. This careful monitoring helped ensure the intervention was delivered consistently and in accordance with the established guidelines. During the study period, fidelity assessment was performed among 18 cases, totaling 72 sessions and the outcome was very satisfactory **(eTable 1 of Supplement 2).**

eReference

Hui, C. L. M., Suen, Y. N., Lam, B. Y. H., Wong, S. M. Y., Wong, C. S. M., Lui, S. S. Y., ... & Chen, E. Y. H. (2022). LevelMind@ JC: Development and evaluation of a community early intervention program for young people in Hong Kong. *Early Intervention in Psychiatry*, *16* (8), 920-925.
Hayati Rezvan P, Lee KJ, Simpson JA. (2015). The rise of multiple imputation: a review of the reporting and implementation of the method in medical research. *BMC Med Res Methodol,* 15, 30.

© 2025 Suen YN et al. *JAMA Network Open*.

eResults. Supplementary Results

Subgroup Analyses

Distress level. The LiON group demonstrated greater improvement in resilience ($F_{(1,59)} = 6.09$, $p = 0.02$, $\eta_p^2 = 0.09$), general stress level ($F_{(1,59)} = 10.43$, $p = 0.002$, $\eta_p^2 = 0.15$) and overall negative emotion ($F_{(1,59)} = 5.34$, $p = 0.02$, $\eta_p^2 = 0.08$), only among those with lower initial distress level (K6 <11) (**eTable 6a**).

Sex. The LiON group demonstrated greater improvement in subjective sleep quality ($F_{(1,84)} = 4.14$, $p = 0.045$, $\eta_p^2 = 0.05$), self-efficacy ($F_{(1,84)} = 4.56$, $p = 0.04$, $\eta_p^2 = 0.05$), anxiety symptoms ($F_{(1,84)} = 4.50$, $p = 0.04$, $\eta_p^2 = 0.05$), general stress levels ($F_{(1,84)} = 7.07$, $p = 0.009$, $\eta_p^2 = 0.08$), and overall negative emotion ($F_{(1,84)} = 6.12$, $p = 0.02$, $\eta_p^2 = 0.07$), only among female (**eTable 7**). With no significant interaction effect seen, we should note that the male subgroup had a very small sample size (n = 33).

Age. The LiON group demonstrated greater improvement in resilience ($F_{(1,45)} = 7.41$, $p = 0.009$, $\eta_p^2 = 0.14$), and overall negative emotion ($F_{(1,46)} = 4.56$, $p = 0.04$, $\eta_p^2 = 0.09$), only among younger participants (**eTable 8**).

Modules. The LiON group demonstrated greater improvement in resilience ($F_{(1,68)} = 9.32$, $p = 0.003$, $\eta_p^2 = 0.12$) among participants who received the stress coping module (**eTable 9**). With no significant interaction effect seen, we should note that the problem solving and sleep relaxation and management subgroups each had a very small sample size (n = 11 and 37, respectively).

© 2025 Suen YN et al. *JAMA Network Open*.

eTable 3. Statistics for the Intention-to-Treat Comparison Between the LiON and WL Groups on All Outcomes

Outcomes	Time points or p-values	LiON (n = 62)	WL (n = 58)	*P* (LiON vs. WL)	Interaction effects				
		Mean (SD)	Mean (SD)			*F*	df1, df2	*P*	η_p^2
**Primary Outcomes**									
Depressive symptoms (DASS-D)	T_0	16.45 (8.95)	16.66 (9.57)	0.91					
	T_1	9.68 (9.24)	11.83 (8.99)	0.20	Group x Time (T_0 – T_1)	1.86	1,116	0.17	0.02
	T_2	10.71 (9.60)	10.79 (8.24)	0.97	Group x Time (T_1 – T_2)	3.71	1,116	0.06	0.03
	P (T_0 *vs.* T_1)	**<0.001**	**<0.001**						
	P (T_1 *vs.* T_2)	0.17	0.18						
Anxiety symptoms (DASS-A)	T_0	15.16 (8.44)	13.72 (7.61)	0.31					
	T_1	9.13 (7.85)	10.21 (5.91)	0.42	Group x Time (T_0 – T_1)	3.26	1,116	0.07	0.03
	T_2	9.58 (8.16)	8.97 (6.17)	0.64	Group x Time (T_1 – T_2)	3.42	1,116	0.07	0.03
	P (T_0 *vs.* T_1)	**<0.001**	**<0.001**						
	P (T_1 *vs.* T_2)	0.48	0.06						
Overall psychological distress (K6)	T_0	10.56 (4.55)	10.34 (4.37)	0.75					
	T_1	6.82 (4.64)	7.40 (4.30)	0.49	Group x Time (T_0 – T_1)	1.41	1,116	0.24	0.01
	T_2	6.45 (4.88)	6.66 (5.06)	0.83	Group x Time (T_1 – T_2)	0.42	1,116	0.52	0.004
	P (T_0 *vs.* T_1)	**<0.001**	**<0.001**						
	P (T_1 *vs.* T_2)	0.35	0.07						
**Secondary outcomes**									
General stress level (DASS-S)	T_0	22.45 (7.80)	19.86 (7.42)	0.051					
	T_1	14.97 (8.61)	15.48 (7.70)	0.76	Group x Time (T_0 – T_1)	**5.54**	**1,116**	**0.02**	**0.05**
	T_2	13.94 (9.05)	14.24 (8.15)	0.88	Group x Time (T_1 – T_2)	0.03	1,116	0.86	0.000
	P (T_0 *vs.* T_1)	**<0.001**	**<0.001**						
	P (T_1 *vs.* T_2)	0.21	0.15						
Overall negative emotion (DASS Total)	T_0	54.06 (21.14)	50.24 (20.57)	0.29					
	T_1	33.77 (23.76)	37.52 (18.88)	0.36	Group x Time (T_0 – T_1)	**4.72**	**1,116**	**0.03**	**0.04**
	T_2	34.23 (25.11)	34.00 (19.22)	0.94	Group x Time (T_1 – T_2)	2.19	1,116	0.14	0.02
	P (T_0 *vs.* T_1)	**<0.001**	**<0.001**						
	P (T_1 *vs.* T_2)	0.81	0.07						
Subjective sleep quality	T_0	2.66 (0.51)	2.57 (0.68)	0.34					
	T_1	2.34 (0.68)	2.43 (0.65)	0.48	Group x Time (T_0 – T_1)	2.35	1,116	0.13	0.02
	T_2	2.32 (0.72)	2.33 (0.63)	>0.99	Group x Time (T_1 – T_2)	0.59	1,116	0.44	0.01
	P (T_0 *vs.* T_1)	**<0.001**	0.12						
	P (T_1 *vs.* T_2)	0.84	0.21						
Resilience (BRE)	T_0	3.70 (0.53)	3.65 (0.56)	0.58					
	T_1	3.22 (0.64)	3.44 (0.56)	0.06	Group x Time (T_0 – T_1)	**6.24**	**1,116**	**0.01**	**0.05**
	T_2	3.13 (0.65)	3.20 (0.62)	0.59	Group x Time (T_1 – T_2)	3.34	1,116	0.07	0.03
	P (T_0 *vs.* T_1)	**<0.001**	**0.007**						
	P (T_1 *vs.* T_2)	0.13	**<0.001**						
Self-efficacy	T_0	3.19 (0.50)	3.11 (0.53)	0.39					
	T_1	2.77 (0.54)	2.81 (0.59)	0.70	Group x Time (T_0 – T_1)	1.32	1,116	0.25	0.01
	T_2	2.57 (0.54)	2.57 (0.65)	0.96	Group x Time (T_1 – T_2)	0.23	1,116	0.63	0.002
	P (T_0 *vs.* T_1)	**<0.001**	**<0.001**						
	P (T_1 *vs.* T_2)	**0.003**	**<0.001**						
HRQoL (SF6D)	T_0	0.68 (0.11)	0.70 (0.10)	0.30					
	T_1	0.75 (0.11)	0.75 (0.09)	0.92	Group x Time (T_0 – T_1)	0.89	1,116	0.35	0.01
	T_2	0.76 (0.10)	0.77 (0.10)	0.41	Group x Time (T_1 – T_2)	0.57	1,116	0.45	0.005
	P (T_0 *vs.* T_1)	**<0.001**	**<0.001**						
	P (T_1 *vs.* T_2)	0.57	0.11						

Note. LiON, low-intensity online intervention; WL, waitlist control; SD, standard deviation; DASS-D, Depression subscale of the Depression, Anxiety, and Stress Scale; DASS-A, Anxiety subscale of the Depression, Anxiety, and Stress Scale; DASS-S, Stress subscale of the Depression, Anxiety, and Stress Scale-Stress subscale; K6, Kessler Psychological Distress Scale 6-item version; HRQoL, health-related quality of life; SF6D, SF6D index derived from 12-Item Short Form Survey.

eTable 4. Statistics for the Per-Protocol Comparison Between the LiON and WL Groups on All Outcomes

**Outcomes**	**Time points or p-values**	**LiON (n = 55)**	**WL (n = 47)**	***P* (LiON vs. WL)**	**Interaction effects**				
		**Mean (SD)**	**Mean (SD)**			***F***	**df1, df2**	***P***	η_p^2
**Primary outcomes**									
Depressive symptoms (DASS-D)	T_0	15.93 (8.49)	16.51 (9.39)	0.75					
	T_1	8.47 (8.19)	11.11 (8.46)	0.12					
	T_2	9.64 (8.79)	9.83 (7.3)	0.91					
	P (T_0 *vs.* T_1)	**<0.001**	**<0.001**		Group x Time (T_0 – T_1)	1.53	1,98	0.22	0.02
	P (T_1 *vs.* T_2)	0.18	0.17		Group x Time (T_1 – T_2)	3.71	1,98	0.06	0.04
Anxiety symptoms (DASS-A)	T_0	14.65 (7.98)	14.21 (7.96)	**<0.001**					
	T_1	7.85 (6.69)	10.13 (5.93)	0.77					
	T_2	8.36 (7.19)	8.6 (6.2)	0.08					
	P (T_0 *vs.* T_1)	**<0.001**	0.48		Group x Time (T_0 – T_1)	2.95	1,98	0.09	0.03
	P (T_1 *vs.* T_2)	**<0.001**	**0.001**		Group x Time (T_1 – T_2)	3.76	1,98	0.06	0.04
Overall psychological distress (K6)	T_0	10.33 (4.3)	10.15 (4.3)	0.83					
	T_1	6.29 (4.18)	6.94 (3.76)	0.42					
	T_2	5.87 (4.43)	6.02 (4.68)	0.87					
	P (T_0 *vs.* T_1)	**<0.001**	**<0.001**		Group x Time (T_0 – T_1)	1.15	1,98	0.29	0.01
	P (T_1 *vs.* T_2)	0.36	0.07		Group x Time (T_1 – T_2)	0.56	1,98	0.46	0.01
**Secondary outcomes**									
General stress level (DASS-S)	T_0	21.82 (7.27)	19.79 (7.33)	0.15					
	T_1	13.78 (7.6)	14.38 (6.8)	0.68					
	T_2	12.62 (7.97)	12.85 (7.15)	0.88					
	P (T_0 *vs.* T_1)	**<0.001**	**<0.001**		Group x Time (T_0 – T_1)	3.19	1,98	0.08	0.03
	P (T_1 *vs.* T_2)	0.22	0.13		Group x Time (T_1 – T_2)	0.07	1,98	0.79	0.00
Overall negative emotion (DASS Total)	T_0	52.4 (19.24)	50.51 (20.51)	0.62					
	T_1	30.11 (20.38)	35.62 (17.65)	0.16					
	T_2	30.62 (22.22)	31.28 (17.52)	0.87					
	P (T_0 *vs.* T_1)	**<0.001**	**<0.001**		Group x Time (T_0 – T_1)	3.50	1,98	0.06	0.03
	P (T_1 *vs.* T_2)	0.81	0.06		Group x Time (T_1 – T_2)	2.43	1,98	0.12	0.02
Subjective sleep quality	T_0	2.62 (0.49)	2.53 (0.72)	0.45					
	T_1	2.29 (0.66)	2.4 (0.68)	0.39					
	T_2	2.27 (0.71)	2.28 (0.65)	0.98					
	P (T_0 *vs.* T_1)	**0.001**	0.21		Group x Time (T_0 – T_1)	2.12	1,98	0.15	0.02
	P (T_1 *vs.* T_2)	0.84	0.20		Group x Time (T_1 – T_2)	0.68	1,98	0.41	0.01
Resilience (BRE)	T_0	3.72 (0.52)	3.62 (0.59)	0.32					
	T_1	3.20 (0.64)	3.40 (0.58)	0.10					
	T_2	3.10 (0.64)	3.11 (0.63)	0.96					
	P (T_0 *vs.* T_1)	**<0.001**	**0.021**		**Group x Time (T_0 – T_1)**	**6.26**	**1,98**	**0.01**	**0.06**
	P (T_1 *vs.* T_2)	0.128	**<0.001**		**Group x Time (T_1 – T_2)**	**4.30**	**1,98**	**0.04**	**0.04**
Self-efficacy	T_0	3.20 (0.49)	3.12 (0.55)	0.41					
	T_1	2.72 (0.52)	2.77 (0.57)	0.66					
	T_2	2.50 (0.49)	2.47 (0.61)	0.77					
	P (T_0 *vs.* T_1)	**<0.001**	**<0.001**		Group x Time (T_0 – T_1)	1.26	1,98	0.26	0.01
	P (T_1 *vs.* T_2)	**0.003**	**<0.001**		Group x Time (T_1 – T_2)	0.57	1,98	0.45	0.01
HRQoL (SF6D)	T_0	0.69 (0.11)	0.70 (0.11)	0.39					
	T_1	0.77 (0.1)	0.76 (0.09)	0.84					
	T_2	0.77 (0.09)	0.79 (0.09)	0.48					
	P (T_0 *vs.* T_1)	**<0.001**	**<0.001**		Group x Time (T_0 – T_1)	1.20	1,98	0.28	0.01
	P (T_1 *vs.* T_2)	**<0.001**	**<0.001**		Group x Time (T_1 – T_2)	0.68	1,98	0.41	0.01

Note. LiON, low-intensity online intervention; WL, waitlist control; SD, standard deviation; DASS-D, Depression subscale of the Depression, Anxiety, and Stress Scale; DASS-A, Anxiety subscale of the Depression, Anxiety, and Stress Scale; DASS-S, Stress subscale of the Depression, Anxiety, and Stress Scale-Stress subscale; K6, Kessler Psychological Distress Scale 6-item version; HRQoL, health-related quality of life; SF6D, SF6D index derived from 12-Item Short Form Survey.

© 2025 Suen YN et al. *JAMA Network Open*.

eTable 5. Statistics for the Multiple Imputation Comparison Between the LiON and WL Groups on All Outcomes

Outcomes	Time points or p-values	LiON (n = 55)	WL (n = 47)	*P* (LiON vs. WL)	Interaction effects				
		Mean (SD)	Mean (SD)			*F*	df1, df2	*P*	η_p^2
**Primary outcomes**									
Depressive symptoms (DASS-D)	T_0	16.45 (8.95)	16.66 (9.57)	0.92					
	T_1	8.77 (8.57)	11.86 (9.57)	0.07					
	T_2	10.03 (8.92)	9.66 (7.73)	0.82					
	P (T_0 *vs.* T_1)	**<0.001**	**<0.001**		Group x Time (T_0 – T_1)	1.21	1,116	0.27	0.010
	P (T_1 *vs.* T_2)	0.22	**0.04**		Group x Time (T_1 – T_2)	0.95	1,116	0.33	0.008
Anxiety symptoms (DASS-A)	T_0	15.16 (8.44)	13.72 (7.61)	0.31					
	T_1	8.26 (7.13)	10.17 (6.26)	0.13					
	T_2	8.87 (7.57)	8.03 (6.12)	0.51					
	P (T_0 *vs.* T_1)	**<0.001**	**0.001**		Group x Time (T_0 – T_1)	0.03	1,116	0.86	0.000
	P (T_1 *vs.* T_2)	0.46	**0.01**		Group x Time (T_1 – T_2)	0.22	1,116	0.64	0.002
Overall psychological distress (K6)	T_0	10.56 (4.55)	10.34 (4.37)	0.75					
	T_1	6.18 (4.38)	7.09 (4.47)	0.25					
	T_2	5.24 (4.54)	5.19 (4.70)	0.97					
	P (T_0 *vs.* T_1)	**<0.001**	**<0.001**		Group x Time (T_0 – T_1)	0.23	1,116	0.63	0.002
	P (T_1 *vs.* T_2)	0.11	**0.02**		Group x Time (T_1 – T_2)	0.42	1,116	0.52	0.004
General stress level (DASS-S)	T_0	22.45 (7.80)	19.86 (7.42)	0.05					
	T_1	13.87 (8.24)	15.17 (8.20)	0.40					
	T_2	12.65 (7.60)	12.69 (7.05)	0.98					
	P (T_0 *vs.* T_1)	**<0.001**	**<0.001**		Group x Time (T_0 – T_1)	0.33	1,116	0.57	0.003
	P (T_1 *vs.* T_2)	0.25	**0.03**		Group x Time (T_1 – T_2)	0.30	1,116	0.59	0.003
Overall negative emotion (DASS Total)	T_0	54.06 (21.14)	50.24 (20.57)	0.29					
	T_1	30.97 (21.65)	37.07 (19.59)	0.11					
	T_2	31.48 (21.72)	30.28 (16.97)	0.74					
	P (T_0 *vs.* T_1)	**<0.001**	**<0.001**		Group x Time (T_0 – T_1)	0.10	1,116	0.75	0.001
	P (T_1 *vs.* T_2)	0.83	**0.006**		Group x Time (T_1 – T_2)	0.55	1,116	0.46	0.005
Subjective sleep quality	T_0	2.66 (0.51)	2.57 (0.68)	0.34					
	T_1	2.23 (0.66)	2.31 (0.71)	0.51					
	T_2	2.18 (0.80)	2.12 (0.73)	0.66					
	P (T_0 *vs.* T_1)	**<0.001**	**0.01**		Group x Time (T_0 – T_1)	0.01	1,116	0.91	0.000
	P (T_1 *vs.* T_2)	0.60	**0.047**		Group x Time (T_1 – T_2)	0.01	1,116	0.93	0.000
Resilience (BRE)	T_0	3.70 (0.53)	0.65 (0.56)	0.58					
	T_1	3.16 (0.67)	3.34 (0.61)	0.12					
	T_2	3.22 (0.71)	3.32 (0.73)	0.46					
	P (T_0 *vs.* T_1)	**<0.001**	**<0.001**		Group x Time (T_0 – T_1)	0.54	1,116	0.46	0.005
	P (T_1 *vs.* T_2)	0.47	0.87		Group x Time (T_1 – T_2)	1.84	1,16	0.18	0.016
Self-efficacy	T_0	3.19 (0.50)	3.11 (0.53)	0.39					
	T_1	2.67 (0.59)	2.70 (0.62)	0.79					
	T_2	2.38 (0.58)	2.29 (0.68)	0.47					
	P (T_0 *vs.* T_1)	**<0.001**	**<0.001**		Group x Time (T_0 – T_1)	0.10	1,116	0.75	0.001
	P (T_1 *vs.* T_2)	**<0.001**	**<0.001**		Group x Time (T_1 – T_2)	0.08	1,116	0.78	0.001
HRQoL (SF6D)	T_0	0.68 (0.11)	0.70 (0.10)	0.30					
	T_1	0.76 (0.11)	0.76 (0.09)	0.79					
	T_2	0.76 (0.10)	0.78 (0.10)	0.30					
	P (T_0 *vs.* T_1)	**<0.001**	**<0.001**		Group x Time (T_0 – T_1)	0.23	1,116	0.64	0.002
	P (T_1 *vs.* T_2)	0.92	0.17		Group x Time (T_1 – T_2)	0.24	1,116	0.63	0.002

Note. LiON, low-intensity online intervention; WL, waitlist control; SD, standard deviation; DASS-D, Depression subscale of the Depression, Anxiety, and Stress Scale; DASS-A, Anxiety subscale of the Depression, Anxiety, and Stress Scale; DASS-S, Stress subscale of the Depression, Anxiety, and Stress Scale-Stress subscale; K6, Kessler Psychological Distress Scale 6-item version; HRQoL, health-related quality of life; SF6D, SF6D index derived from 12-Item Short Form Survey.

eTable 6. Subgroup Analysis by Distress Levels on the Intention-to-Treat Comparison Between the LiON and WL Groups on All Outcomes

Outcomes	Time points or p-values	K6 < 11							K6 ≥ 11						
		LiON (n = 31)	WL (n = 32)	*P* (LiON vs. WL)	Group x Time ($T_0 – T_1$) interaction effects				LiON (n = 31)	WL (n = 26)	*P* (LiON vs. WL)	Group x Time ($T_0 – T_1$) interaction effects			
		Mean (SD)	Mean (SD)		*F*	df1, df2	*P*	η_p^2	Mean (SD)	Mean (SD)		*F*	df1, df2	*P*	η_p^2
**Primary outcomes**															
Depressive symptoms (DASS-D)	T_0	11.10 (6.43)	10.94 (5.75)	>0.99	0.60	1,59	0.44	0.01	21.81 (7.89)	23.69 (8.63)	0.41	0.73	1,53	0.40	0.01
	T_1	6.84 (7.23)	8.13 (6.09)	0.42					12.52 (10.24)	16.38 (9.96)	0.17				
	P (T_0 *vs.* T_1)	**0.002**	**0.03**						**<0.001**	**<0.001**					
Anxiety symptoms (DASS-A)	T_0	11.87 (6.11)	10.31 (6.59)	0.27	3.88	1,59	0.054	0.06	18.45 (9.22)	17.92 (6.70)	0.82	0.38	1,53	0.54	0.01
	T_1	6.97 (6.00)	8.56 (6.04)	0.32					11.29 (8.92)	12.23 (5.16)	0.64				
	P (T_0 *vs.* T_1)	**<0.001**	0.16						**<0.001**	**0.002**					
Overall psychological distress (K6)	T_0	6.90 (2.29)	7.19 (2.28)	0.65	0.67	1,59	0.42	0.01	14.23 (3.03)	14.23 (2.94)	0.98	0.35	1,53	0.56	0.01
	T_1	4.74 (3.2)	5.63 (2.73)	0.22					8.90 (4.95)	9.58 (4.89)	0.63				
	P (T_0 *vs.* T_1)	**<0.001**	**0.01**						**<0.001**	**<0.001**					
**Secondary outcomes**															
General stress level (DASS-S)	T_0	19.35 (7.35)	15.44 (5.62)	**0.009**	**10.43**	**1,59**	**0.002**	**0.15**	25.55 (7.08)	25.31 (5.51)	0.91	0.10	1,53	0.75	0.002
	T_1	12.58 (7.25)	13.63 (8.06)	0.65					17.35 (9.3)	17.77 (6.7)	0.83				
	P (T_0 *vs.* T_1)	**<0.001**	0.12						**<0.001**	**<0.001**					
Overall negative emotion (DASS Total)	T_0	42.32 (15.17)	36.69 (13.49)	0.10	**5.34**	**1,59**	**0.02**	**0.08**	65.81 (19.85)	66.92 (14.63)	0.81	0.52	1,53	0.47	0.01
	T_1	26.39 (18.7)	30.31 (16.53)	0.40					41.16 (26.18)	46.38 (18.05)	0.40				
	P (T_0 *vs.* T_1)	**<0.001**	**0.04**						**<0.001**	**<0.001**					
Subjective sleep quality	T_0	2.55 (0.51)	2.44 (0.72)	0.35	0.99	1,59	0.33	0.02	2.77 (0.5)	2.73 (0.6)	0.84	1.29	1,53	0.26	0.02
	T_1	2.26 (0.68)	2.31 (0.59)	0.84					2.42 (0.67)	2.58 (0.7)	0.36				
	P (T_0 *vs.* T_1)	**0.02**	0.35						**0.004**	0.23					
Resilience (BRE)	T_0	3.55 (0.52)	3.46 (0.62)	0.38	**6.09**	**1,59**	**0.02**	**0.09**	3.84 (0.51)	3.88 (0.38)	0.76	0.97	1,53	0.33	0.02
	T_1	3.09 (0.63)	3.34 (0.48)	0.12					3.35 (0.63)	3.56 (0.63)	0.24				
	P (T_0 *vs.* T_1)	**<0.001**	0.25						**<0.001**	**0.01**					
Self-efficacy	T_0	3.00 (0.39)	2.91 (0.55)	0.47	0.62	1,59	0.43	0.01	3.39 (0.53)	3.37 (0.39)	0.84	0.42	1,53	0.52	0.01
	T_1	2.68 (0.48)	2.72 (0.58)	0.86					2.85 (0.59)	2.92 (0.59)	0.64				
	P (T_0 *vs.* T_1)	**0.003**	**0.048**						**<0.001**	**<0.001**					
HRQoL (SF6D)	T_0	0.75 (0.09)	0.74 (0.09)	0.93	0.62	1,59	0.43	0.01	0.62 (0.09)	0.66 (0.1)	0.19	0.17	1,53	0.68	0.003
	T_1	0.79 (0.1)	0.77 (0.09)	0.40					0.72 (0.12)	0.74 (0.1)	0.48				
	P (T_0 *vs.* T_1)	**0.02**	0.19						**<0.001**	**<0.001**					

Note. LiON, low-intensity online intervention; WL, waitlist control; SD, standard deviation; DASS-D, Depression subscale of the Depression, Anxiety, and Stress Scale; DASS-A, Anxiety subscale of the Depression, Anxiety, and Stress Scale; DASS-S, Stress subscale of the Depression, Anxiety, and Stress Scale-Stress subscale; K6, Kessler Psychological Distress Scale 6-item version; HRQoL, health-related quality of life; SF6D, SF6D index derived from 12-Item Short Form Survey.

© 2025 Suen YN et al. *JAMA Network Open*.

eTable 7. Subgroup Analysis by Sex on the Intention-to-Treat Comparison Between the LiON and WL Groups on All Outcomes

Outcomes	Time points or p-values	Female							Male						
		LiON (n = 45)	WL (n = 42)	*P* (LiON vs. WL)	Group x Time (T_0 – T_1) interaction effects				LiON (n = 17)	WL (n = 16)	*P* (LiON vs. WL)	Group x Time (T_0 – T_1) interaction effects			
		Mean (SD)	Mean (SD)		*F*	df1, df2	*P*	η_p^2	Mean (SD)	Mean (SD)		*F*	df1, df2	*P*	η_p^2
**Primary outcomes**															
Depressive symptoms (DASS-D)	T_0	17.2 (8.86)	16.67 (10.44)	0.78	1.97	1,84	0.16	0.02	14.47 (9.15)	16.63 (7.07)	0.46	0.07	1,30	0.80	0.002
	T_1	9.42 (9.45)	11.33 (9.47)	0.34					10.35 (8.89)	13.13 (7.73)	0.35				
	P (T_0 *vs.* T_1)	**<0.001**	**<0.001**						**0.02**	0.050					
Anxiety symptoms (DASS-A)	T_0	15.96 (8.52)	14.38 (8.43)	0.35	**4.50**	**1,84**	**0.04**	**0.05**	13.06 (8.07)	12 (4.62)	0.65	0.06	1,30	0.81	0.002
	T_1	8.31 (7.63)	10.38 (6.2)	0.18					11.29 (8.24)	9.75 (5.21)	0.53				
	P (T_0 *vs.* T_1)	**<0.001**	**0.002**						0.21	0.13					
Overall psychological distress (K6)	T_0	11.13 (4.36)	10.38 (4.34)	0.38	2.99	1,84	0.09	0.03	9.06 (4.84)	10.25 (4.6)	0.48	0.30	1,30	0.59	0.01
	T_1	6.73 (4.47)	7.33 (4.41)	0.53					7.06 (5.19)	7.56 (4.13)	0.76				
	P (T_0 *vs.* T_1)	**<0.001**	**<0.001**						**0.03**	**0.005**					
**Secondary outcomes**															
General stress level (DASS-S)	T_0	23.73 (7.49)	20.43 (7.13)	0.03	**7.07**	**1,84**	**0.009**	**0.08**	19.06 (7.81)	18.38 (8.17)	0.81	0.000	1,30	0.97	0.000
	T_1	14.49 (8.87)	15.43 (7.56)	0.63					16.24 (8)	15.63 (8.3)	0.83				
	P (T_0 *vs.* T_1)	**<0.001**	**<0.001**						0.07	0.09					
Overall negative emotion (DASS Total)	T_0	56.89 (20.48)	51.48 (21.78)	0.21	**6.12**	**1,84**	**0.02**	**0.07**	46.59 (21.67)	47 (17.19)	0.95	0.000	1,30	0.97	0.000
	T_1	32.22 (24.02)	37.14 (19.26)	0.31					37.88 (23.28)	38.5 (18.42)	0.93				
	P (T_0 *vs.* T_1)	**<0.001**	**<0.001**						**0.04**	0.050					
Subjective sleep quality	T_0	2.69 (0.47)	2.6 (0.66)	0.38	**4.14**	**1,84**	**0.045**	**0.05**	2.59 (0.62)	2.5 (0.73)	0.7	0.50	1,30	0.48	0.02
	T_1	2.29 (0.66)	2.5 (0.63)	0.14					2.47 (0.72)	2.25 (0.68)	0.37				
	P (T_0 *vs.* T_1)	**<0.001**	0.40						0.37	0.07					
Resilience (BRE)	T_0	3.79 (0.54)	3.75 (0.53)	0.7	3.35	1,84	0.07	0.04	3.45 (0.45)	3.38 (0.57)	0.68	3.73	1,30	0.06	0.11
	T_1	3.24 (0.66)	3.45 (0.58)	0.14					3.16 (0.59)	3.41 (0.52)	0.21				
	P (T_0 *vs.* T_1)	**<0.001**	**0.002**						**0.02**	0.80					
Self-efficacy	T_0	3.27 (0.5)	3.14 (0.47)	0.25	**4.56**	**1,84**	**0.04**	**0.05**	3 (0.47)	3.03 (0.67)	0.87	1.84	1,30	0.19	0.06
	T_1	2.72 (0.54)	2.87 (0.54)	0.22					2.88 (0.55)	2.66 (0.7)	0.29				
	P (T_0 *vs.* T_1)	**<0.001**	**0.002**						0.38	**0.01**					
HRQoL (SF6D)	T_0	0.67 (0.11)	0.7 (0.11)	0.18	0.40	1,84	0.53	0.005	0.72 (0.1)	0.72 (0.09)	0.79	0.91	1,30	0.35	0.03
	T_1	0.74 (0.12)	0.76 (0.09)	0.5					0.78 (0.09)	0.74 (0.09)	0.27				
	P (T_0 *vs.* T_1)	**<0.001**	**0.002**						**0.01**	0.21					

Note. LiON, low-intensity online intervention; WL, waitlist control; SD, standard deviation; DASS-D, Depression subscale of the Depression, Anxiety, and Stress Scale; DASS-A, Anxiety subscale of the Depression, Anxiety, and Stress Scale; DASS-S, Stress subscale of the Depression, Anxiety, and Stress Scale-Stress subscale; K6, Kessler Psychological Distress Scale 6-item version; HRQoL, health-related quality of life; SF6D, SF6D index derived from 12-Item Short Form Survey.

© 2025 Suen YN et al. *JAMA Network Open*.

eTable 8. Subgroup Analysis by Age on the Intention-to-Treat Comparison Between the LiON and WL Groups on All Outcomes

Outcomes	Time points or p-values	Age < 22 years							Age ≥ 22 years						
		LiON (n = 23)	WL (n = 26)	*P* (LiON vs. WL)	Group x Time ($T_0 - T_1$) interaction effects				LiON (n = 39)	WL (n = 32)	*P* (LiON vs. WL)	Group x Time ($T_0 - T_1$) interaction effects			
		Mean (SD)	Mean (SD)		*F*	df1, df2	*P*	η_p^2	Mean (SD)	Mean (SD)		*F*	df1, df2	*P*	η_p^2
**Primary outcomes**															
Depressive symptoms (DASS-D)	T_0	16.26 (8.14)	15.69 (8.52)	0.83	3.99	1,45	0.051	0.08	16.56 (9.49)	17.44 (10.41)	0.76	0.23	1,45	0.64	0.003
	T_1	8.17 (8.86)	12 (9.26)	0.17					10.56 (9.46)	11.69 (8.92)	0.45				
	P (T_0 *vs.* T_1)	**<0.001**	**0.01**						**<0.001**	**<0.001**					
Anxiety symptoms (DASS-A)	T_0	14.43 (8.61)	12.46 (6.63)	0.42	2.91	1,45	0.09	0.06	15.59 (8.41)	14.75 (8.28)	0.49	1.13	1,45	0.29	0.02
	T_1	7.74 (7.51)	9.77 (6.63)	0.35					9.95 (8.02)	10.56 (5.33)	0.73				
	P (T_0 *vs.* T_1)	**<0.001**	0.07						**<0.001**	**0.007**					
Overall psychological distress (K6)	T_0	9.17 (3.89)	9.88 (4.33)	0.52	1.01	1,45	0.32	0.02	11.38 (4.76)	10.72 (4.44)	0.44	1.20	1,45	0.28	0.02
	T_1	5.57 (4.08)	7.38 (3.87)	0.13					7.56 (4.83)	7.41 (4.68)	0.88				
	P (T_0 *vs.* T_1)	**<0.001**	**<0.001**						**<0.001**	**<0.001**					
**Secondary outcomes**															
General stress level (DASS-S)	T_0	19.65 (7.38)	18.23 (6.13)	0.52	2.93	1,45	0.09	0.06	24.1 (7.66)	21.19 (8.17)	0.11	2.73	1,45	0.10	0.04
	T_1	12.35 (7.57)	14.54 (6.73)	0.27					16.51 (8.91)	16.25 (8.44)	0.97				
	P (T_0 *vs.* T_1)	**<0.001**	**0.01**						**<0.001**	**<0.001**					
Overall negative emotion (DASS Total)	T_0	50.35 (20.89)	46.38 (17.8)	0.53	**4.56**	**1,45**	**0.04**	**0.09**	56.26 (21.25)	53.38 (22.35)	0.48	1.56	1,45	0.22	0.02
	T_1	28.26 (22.2)	36.31 (19.45)	0.2					37.03 (24.33)	38.5 (18.66)	0.68				
	P (T_0 *vs.* T_1)	**<0.001**	**0.007**						**<0.001**	**<0.001**					
Subjective sleep quality	T_0	2.61 (0.58)	2.27 (0.67)	0.08	2.44	1,45	0.13	0.05	2.69 (0.47)	2.81 (0.59)	0.5	0.42	1,45	0.52	0.01
	T_1	2.26 (0.75)	2.23 (0.65)	0.9					2.38 (0.63)	2.59 (0.61)	0.21				
	P (T_0 *vs.* T_1)	**0.02**	0.74						**0.001**	0.10					
Resilience (BRE)	T_0	3.58 (0.53)	3.47 (0.63)	0.58	**7.41**	**1,45**	**0.009**	**0.14**	3.76 (0.53)	3.79 (0.46)	0.55	0.34	1,45	0.56	0.01
	T_1	3.07 (0.6)	3.44 (0.61)	0.04					3.31 (0.65)	3.44 (0.52)	0.31				
	P (T_0 *vs.* T_1)	**<0.001**	**<0.001**						**<0.001**	**<0.001**					
Self-efficacy	T_0	3.15 (0.61)	3.12 (0.62)	0.83	0.39	1,45	0.54	0.01	3.22 (0.43)	3.11 (0.45)	0.38	01.64	1,45	0.20	0.02
	T_1	2.59 (0.51)	2.67 (0.62)	0.64					2.87 (0.53)	2.92 (0.56)	0.64				
	P (T_0 *vs.* T_1)	**<0.001**	**0.001**						**<0.001**	**0.050**					
HRQoL (SF6D)	T_0	0.73 (0.11)	0.71 (0.11)	0.57	0.20	1,45	0.66	0.004	0.66 (0.1)	0.69 (0.1)	0.09	2.83	1,45	0.10	0.04
	T_1	0.77 (0.09)	0.77 (0.08)	0.81					0.74 (0.12)	0.74 (0.1)	0.95				
	P (T_0 *vs.* T_1)	**0.03**	**0.004**						**<0.001**	**0.04**					

Note. LiON, low-intensity online intervention; WL, waitlist control; SD, standard deviation; DASS-D, Depression subscale of the Depression, Anxiety, and Stress Scale; DASS-A, Anxiety subscale of the Depression, Anxiety, and Stress Scale; DASS-S, Stress subscale of the Depression, Anxiety, and Stress Scale-Stress subscale; K6, Kessler Psychological Distress Scale 6-item version; HRQoL, health-related quality of life; SF6D, SF6D index derived from 12-Item Short Form Survey.

© 2025 Suen YN et al. *JAMA Network Open*.

eTable 9. Subgroup Analysis by Modules on the Intention-to-Treat Comparison Between the LiON and WL Groups on All Outcomes

Outcomes	Time points or p-values	Sleep and relaxation							Stress coping							Problem solving						
		LiON (n = 7)	WL (n = 4)	*P*	Group x Time ($T_0 - T_1$) interaction effects				LiON (n = 41)	WL (n = 31)	*P*	Group x Time ($T_0 - T_1$) interaction effects				LiON (n = 14)	WL (n = 23)	*P*	Group x Time ($T_0 - T_1$) interaction effects			
		Mean (SD)	Mean (SD)		*F*	df1, df2	*P*	η_p^2	Mean (SD)	Mean (SD)		*F*	df1, df2	*P*	η_p^2	Mean (SD)	Mean (SD)		*F*	df1, df2	*P*	η_p^2
**Primary outcomes**																						
Depressive symptoms (DASS-D)	T_0	13.71 (9.27)	14.50 (7.72)	0.19	0.14	1,7	0.72	0.02	16.59 (8.42)	16.19 (9.29)	0.76	2.74	1,68	0.10	0.04	17.43 (10.62)	17.65 (10.46)	0.66	0.04	1,33	0.85	0.001
	T_1	10.00 (10.83)	10.50 (10.38)	0.20					9.27 (9.20)	11.61 (8.17)	0.22					10.71 (9.20)	12.35 (10.16)	0.54				
	P	0.05	0.30						**<0.001**	**0.007**						**0.01**	**0.003**					
Anxiety symptoms (DASS-A)	T_0	13.43 (8.92)	17.50 (9.57)	0.25	0.000	1,7	0.99	0.000	15.56 (8.12)	14.77 (7.60)	0.52	2.72	1,68	0.10	0.04	14.86 (9.60)	11.65 (7.07)	0.38	0.57	1,33	0.46	0.02
	T_1	10.29 (8.90)	15.00 (2.58)	**<0.001**					8.88 (7.60)	10.52 (5.66)	0.26					9.29 (8.58)	8.96 (6.32)	0.88				
	P	0.42	**<0.001**						**<0.001**	**0.009**						**0.03**	0.09					
Overall psychological distress (K6)	T_0	8.43 (4.16)	8.25 (1.71)	0.47	0.79	1,7	0.40	0.10	10.76 (4.47)	10.81 (4.09)	0.79	0.85	1,68	0.36	0.01	11.07 (4.98)	10.09 (5.02)	0.78	1.01	1,33	0.32	0.03
	T_1	5.29 (3.25)	6.75 (2.22)	0.13					6.98 (4.87)	7.48 (4.64)	0.57					7.14 (4.66)	7.39 (4.22)	0.69				
	P	**0.02**	0.49						**<0.001**	**<0.001**						**<0.001**	**<0.001**					
**Secondary outcomes**																						
General stress level (DASS-S)	T_0	17.43 (7.98)	17.50 (9.29)	0.39	0.06	1,7	0.82	0.01	22.63 (7.62)	21.55 (7.78)	0.36	2.22	1,68	0.14	0.03	24.43 (7.73)	18.00 (6.30)	0.02	4.07	1,33	0.05	0.11
	T_1	12.00 (8.49)	15.00 (6.83)	0.55					15.22 (8.80)	16.52 (8.16)	0.51					15.71 (8.44)	14.17 (7.28)	0.55				
	P	**0.02**	0.06						**<0.001**	**0.004**						**<0.001**	**<0.001**					
Overall negative emotion (DASS Total)	T_0	44.57 (24.87)	49.50 (17.54)	0.22	0.000	1,7	0.95	0.001	54.78 (19.37)	52.52 (20.42)	0.45	3.53	1,68	0.06	0.05	56.71 (24.49)	47.3 (21.66)	0.41	1.06	1,33	0.31	0.03
	T_1	32.29 (26.62)	40.50 (12.48)	0.28					33.37 (23.94)	38.65 (18.10)	0.26					35.71 (23.51)	35.48 (21.17)	0.98				
	P	**0.03**	0.12						**<0.001**	**0.001**						**<0.001**	**0.002**					
Subjective sleep quality	T_0	2.86 (0.38)	3.00 (0.00)	0.40	0.08	1,7	0.79	0.01	2.61 (0.54)	2.68 (0.70)	0.95	0.58	1,68	0.45	0.01	2.71 (0.47)	2.35 (0.65)	0.14	2.36	1,33	0.13	0.07
	T_1	2.43 (0.53)	3.00 (0.00)	0.78					2.29 (0.68)	2.42 (0.67)	0.43					2.43 (0.76)	2.35 (0.65)	0.82				
	P	0.49	0.41						**0.001**	0.06						0.08	0.83					
Resilience (BRE)	T_0	3.33 (0.84)	3.83 (0.47)	0.08	2.41	1,7	0.16	0.26	3.76 (0.48)	3.73 (0.53)	0.56	**9.32**	**1,68**	**0.003**	**0.12**	3.69 (0.45)	3.50 (0.61)	0.71	0.09	1,33	0.77	0.003
	T_1	3.10 (0.55)	3.71 (0.28)	**<0.001**					3.22 (0.62)	3.57 (0.60)	0.02					3.27 (0.77)	3.21 (0.47)	0.99				
	P	0.49	**<0.001**						**<0.001**	**<0.001**						**0.03**	**0.02**					
Self-efficacy	T_0	3.00 (0.29)	3.13 (0.25)	0.35	1.28	1,7	0.30	0.15	3.21 (0.51)	3.11 (0.50)	0.29	3.50	1,68	0.07	0.05	3.25 (0.55)	3.11 (0.62)	0.65	0.31	1,33	0.58	0.01
	T_1	2.86 (0.24)	3.13 (0.48)	**<0.001**					2.70 (0.62)	2.85 (0.65)	0.37					2.93 (0.33)	2.70 (0.52)	0.2				
	P	0.58	**<0.001**						**<0.001**	**0.02**						0.06	**0.002**					
HRQoL (SF6D)	T_0	0.73 (0.12)	0.71 (0.13)	0.57	0.000	1,7	>0.99	0.000	0.68 (0.12)	0.69 (0.10)	0.28	0.54	1,68	0.46	0.01	0.68 (0.09)	0.72 (0.11)	0.49	0.67	1,33	0.42	0.02
	T_1	0.74 (0.12)	0.76 (0.10)	0.53					0.76 (0.11)	0.77 (0.08)	0.68					0.74 (0.12)	0.74 (0.10)	0.75				
	P	0.11	0.26						**<0.001**	**<0.001**						0.11	0.46					

Note. LiON, low-intensity online intervention; WL, waitlist control; SD, standard deviation; DASS-D, Depression subscale of the Depression, Anxiety, and Stress Scale; DASS-A, Anxiety subscale of the Depression, Anxiety, and Stress Scale; DASS-S, Stress subscale of the Depression, Anxiety, and Stress Scale-Stress subscale; K6, Kessler Psychological Distress Scale 6-item version; HRQoL, health-related quality of life; SF6D, SF6D index derived from 12-Item Short Form Survey.

© 2025 Suen YN et al. *JAMA Network Open*.
