# Supplementary material for: Low-Intensity Online Intervention for Mental Distress Among Help-Seeking Young People in Hong Kong: A Randomized Clinical Trial
Source: JAMA Netw Open. 2025 Jan 15;8(1):e2454675. doi: 10.1001/jamanetworkopen.2024.54675 (PMC11736507; doi:10.1001/jamanetworkopen.2024.54675)
Supplement: Supplement 3. — Data Sharing Statement [file jamanetwopen-e2454675-s003.pdf]

Data Sharing Statement

Suen. Low-Intensity Online Intervention for Mental Distress Among Help-Seeking Young People in Hong Kong. *JAMA Netw Open*. Published January 15, 2025. doi:10.1001/jamanetworkopen.2024.54675

Data
Additional Information: ClinicalTrials.gov NCT05510453
Data available: No
